# 7T MRI Versus 3T MRI of the Brain in Professional Fighters and Patients With Head Trauma

**DOI:** 10.1089/neur.2023.0001

**Published:** 2023-05-18

**Authors:** Jonathan K. Lee, Charles Bernick, Steve Stephen, Aaron Ritter, Jennifer Bullen, Arvindpaul Mangat, Jennifer Joyce, Stephen E. Jones

**Affiliations:** ^1^ Imaging Institute, Cleveland Clinic, Cleveland, Ohio, USA.; ^2^ Neurological Institute, Lou Ruvo Center for Brain Health, Cleveland Clinic, Cleveland, Ohio, USA.; ^3^ University of Rochester Medical School, Rochester, New York, USA.; ^4^ Hoag's Pickup Family Neurosciences Institute, Hoag Hospital, Newport Beach, California, USA.; ^5^ Quantitative Health Sciences, Cleveland Clinic, Cleveland, Ohio, USA.; ^6^ Department of Medical Imaging, St. Joseph's Health Care London, London, Ontario, Canada.; ^7^ Department of Radiology, University of Cincinnati, Cincinnati, Ohio, USA.

**Keywords:** 7T MRI, professional fighters, repetitive head injury, head trauma

## Abstract

Many studies have investigated the imaging sequelae of repetitive head trauma with mixed results, particularly with regard to the detection of intracranial white matter changes (WMCs) and cerebral microhemorrhages (CMHs) on ≤3 Tesla (T) field magnetic resonance imaging (MRI). 7T MRI, which has recently been approved for clinical use, is more sensitive at detecting lesions associated with multiple neurological diagnoses. In this study, we sought to determine whether 7T MRI would detect more WMCs and CMHs than 3T MRI in 19 professional fighters, 16 patients with single TBI, versus 82 normal healthy controls (NHCs). Fighters and patients with TBI underwent both 3T and 7T MRI; NHCs underwent either 3T (*n* = 61) or 7T (*n* = 21) MRI. Readers agreed on the presence/absence of WMCs in 88% (84 of 95) of 3T MRI studies (Cohen's kappa, 0.76) and in 93% (51 of 55) of 7T MRI studies (Cohen's kappa, 0.79). Readers agreed on the presence/absence of CMHs in 96% (91 of 95) of 3T MRI studies (Cohen's kappa, 0.76) and in 96% (54 of 56) of 7T MRI studies (Cohen's kappa, 0.88). The number of WMCs detected was greater in fighters and patients with TBI than NHCs at both 3T and 7T. Moreover, the number of WMCs was greater at 7T than at 3T for fighters, patients with TBI, and NHCs. There was no difference in the number of CMHs detected with 7T MRI versus 3T MRI or in the number of CMHs observed in fighters/patients with TBI versus NHCs. These initial findings suggest that fighters and patients with TBI may have more WMCs than NHCs and that the improved voxel size and signal-to-noise ratio at 7T may help to detect these changes. As 7T MRI becomes more prevalent clinically, larger patient populations should be studied to determine the cause of these WMCs.

## Introduction

Professional fighters participating in boxing, mixed martial arts (MMA), and martial arts sustain repetitive head impacts (RHIs) over time. Exposure to RHI is thought to be a risk factor for chronic traumatic encephalopathy (CTE).^1–3^ However, because CTE is currently a pathological diagnosis, many clinicians use magnetic resonance imaging (MRI) to monitor for structural changes in the brain; these findings may include reduced brain volumes in various structures (e.g., corpus callosum, thalamus, or hippocampus) and increased incidence of cavum septum pellucidum.^4^

Non-specific white matter changes (WMCs) are defined as focal areas of fluid-attenuated inversion recovery (FLAIR) hyperintensity relative to the adjacent white matter that are not associated with another abnormality, such as a microhemorrage or encephalomalacia. Detection of WMCs and cerebral microhemorrhages (CMHs) on MRI in patients with RHI has been a matter of debate. Whereas some studies have demonstrated greater numbers of WMCs in the brains of contact sport athletes than in normal healthy control (NHC) participants,^5,6^ others have demonstrated contradictory findings,^7,8^ leading some researchers to suggest that the extent of white matter damage is beyond the detection capability of routine 3 Tesla (T) or lower field MRI.^9^ Complicating the picture further, several studies have found that WMCs are likely to be subcortical rather than deep/periventricular in patients with mild head trauma (TBI).^7–10^ In regard to CMHs, some studies have found that these hemorrhages are more common in fighters than in NHCs, but this trend did not reach statistical significance.^9,11,12^ However, a more recent study did demonstrate a significantly increased incidence of CMHs in adolescent football players.^13^

In recent years, 7T MRI has been increasingly used to achieve greater accuracy in neurological diagnoses,^14^ and this field strength was approved by the U.S. Food and Drug Administration for clinical use in 2017. The increased signal from 7T scanners provides greater signal-to-noise ratio, smaller voxel size, increased tissue contrast differences, and greater sensitivity to susceptibility effects on susceptibility-weighted imaging (SWI). In practice, 7T scanners have been shown to be more sensitive than 3T scanners to neurological lesions associated with epilepsy, multiple sclerosis, and other neurological syndromes.^14–16^

Given that previous contradictory findings regarding CMHs and WMCs were demonstrated in studies performed at field strengths of 3T or less, and that there is potentially increased sensitivity to CMHs and WMCs with 7T, we performed this study to determine whether fighters (vs. NHC participants) demonstrate more CMHs and WMCs on 7T MRI than on 3T MRI. We also compared both 3T and 7T imaging results from fighters with RHI to those from a cohort of patients with TBI from a single traumatic event to determine where there are differences between these groups in the number of CMHs and WMCs. Last, the number of CMHs and WMCs detected in fighters on 7T MRI was assessed in relation to various neuropsychiatric scores and the presence or absence of neurofilament light protein, a biomarker of axonal damage.

## Methods

### Study participants

This study was approved by the local institutional review board (IRB; #10-944), and written informed consent was obtained from all study participants. The professional fighters included in this analysis are part of an ongoing longitudinal observational study known as the PABHS (Professional Athletes Brain Health Study).^17^ All fighters in the overall study obtained 3T MRIs, but we recruited volunteers for 7T MRIs until we obtained 20 fighters to participate in the current analysis. One of these fighters was unable to complete the entire 7T MRI scan and was excluded from the study, leaving a final population of 19 fighters. A total of 16 patients with TBI were also included; these patients were all the TBI patients who suffered a single TBI enrolled in a separate 7T neuroimaging registry, which enrolled many different groups of patients with IRB approval (IRB #13-1545). In addition, 61 NHC participants as part of the PABHS who underwent 3T MRI, along with 21 NHC participants who underwent 7T MRI as part of the neuroimaging registry, were included in the analysis. These control participants were screened for any major neurological diseases and a history of major trauma.

### Magnetic resonance imaging protocols

For 3T MRI, a Verio 3T scanner (Siemens Medical Systems, Erlangen, Germany) was used. The protocol included an axial turbo spin-echo (TSE) FLAIR sequence (voxel size = 0.8 × 0.8 × 4 mm; repetition time [TR]/echo time [TE]/inversion time [TI] = 7000/81/2220 ms; 38 sections; scan time = 2 min 36 sec) and axial SWI sequence (voxel size = 0.9 × 0.9 × 0.9 mm; TR/TE = 20/27 ms; 36 sections; scan time = 1 min 17 sec).

For 7T MRI, a 7T research whole-body scanner (MAGNETOM; Siemens Medical Systems) was used with an Agilent 830AS magnet and a single-transmit and 32-channel receive-only phased-array head-only coil (Nova Medical, Wilmington, MA). The protocol included an axial two-dimensional TSE T2 FLAIR sequence (TR/TE = 9000/124 ms; field of view [FOV] = 192 × 192 mm^2^; slice number = 45; slice thickness = 2 mm; in-plane resolution = 0.75 × 0.75 mm^2^; TI = 2600 ms; matrix = 256 × 256; generalized autocalibrating partially parallel acquisitions (GRAPPA) 3; pixel bandwidth 244 Hz/pixel; scan time = 3 min 2 sec) and an axial three-dimensional SWI sequence (TR/TE = 23/15 ms; FOV = 220 × 220 mm^2^; slice number = 144; resolution = 0.49 × 0.49 × 0.8 mm^3^; flip angle = 20 degrees; matrix = 448 × 448; GRAPPA 2; 6/8 partial acquisition in the kz encoding direction; pixel bandwidth = 120 Hz/pixel; scan time = 8 min 16 sec).

### Image interpretation

MRI scans for all study participants were examined by two board-certified neuroradiologists with 6 years (J.L.) and 15 years (S.J.) of experience.

WMCs were defined as focal areas of FLAIR hyperintensity relative to the adjacent white matter. They were recorded as subcortical or periventricular/deep WMCs. WMC foci were excluded if they were <3 mm or if they were associated with another abnormality, such as an area of encephalomalacia. Note that 1 patient with TBI did not undergo axial FLAIR imaging at 7T, so no WMCs were recorded for this patient.

CMHs were defined as clear areas of abnormal susceptibility hypointensity that were focal, rounded, <5 mm, not on the pial or ependymal surface, and not caused by calcification, lacunae, normal vascular structures, or normal variant anomalies such as developmental venous anomalies. Cavernous malformations, with characteristic features of T2 central hyperintensity with a T2 hypointense rim and blooming on SWI, were also excluded.

### Additional testing

All fighters underwent neuropsychological testing, with assessments including central nervous system vital signs (tests of verbal memory, processing speed, psychomotor speed, and reaction time), the Patient Health Questionnaire depression scale, and the Barratt Impulsiveness Scale. For most of the fighters (16 of 19), plasma measures of neurofilament light protein, a marker of axonal damage, were also obtained.

### Statistical analysis

Inter-reader agreement on the presence/absence of WMCs and presence/absence of CMHs was summarized using percent agreement and Cohen's kappa. Median absolute discrepancy in the number of WMCs and CMHs was also reported.

The Wilcoxon signed-rank test was used to assess differences between 3T and 7T MRI with respect to the numbers of WMCs and CMHs detected. These tests were performed separately for fighters and for patients with TBI.

For the 3T MRI data, the Wilcoxon rank-sum test was used to assess group differences (i.e., fighters vs. NHCs and fighters vs. patients with TBI) with respect to the number of WMCs and the number of CMHs. This test was performed separately for fighters and for patients with TBI.

For the 7T MRI data, the Wilcoxon rank-sum test was used to assess group differences (i.e., fighters vs. NHCs and fighters vs. patients with TBI) with respect to the number of WMCs overall, subcortical WMCs, periventricular WMCs, and CMHs. The Wilcoxon signed-rank test was used to assess the null hypothesis that the number of WMCs detected within a fighter or patient with TBI was the same in the subcortical region as in the periventricular region. This test was performed separately for fighters and for patients with TBI.

Among fighters, Spearman's rank correlation was used to assess the association between the number of WMCs detected and various patient characteristics (including neuropsychological test findings). These associations were assessed separately for 3T and 7T.

Because of the presence of some extreme values in the number of WMCs detected, non-parametric tests were selected to avoid distributional assumptions. A significance level of 0.05 was used for all hypothesis tests. All analyses were performed using R software (version 4.0.3; R Foundation for Statistical Computing, Vienna, Austria).

## Results

### Demographics

This sample consisted of 117 participants (19 fighters, 16 patients with TBI, and 82 NHCs). All fighters and patients with TBI were scanned on both the 3T and 7T MRI scanners. NHCs were scanned on only one machine; 61 were scanned on the 3T MRI scanner (3T NHCs) and 21 were scanned on the 7T MRI scanner (7T NHCs). All fighters were male, whereas 52% of 7T NHCs, 92% of 3T NHCs, and 25% of patients with TBI were male. Mean age at the time of scanning was 40 years among fighters, 35 among 7T NHCs, 31 among 3T NHCs, and 30 among patients with TBI (Table 1). Four fighters were active and 15 were retired (Table 2).

**Table 1.** Participant Characteristics

Characteristic	Fighters (*n* = 19)	Patients with TBI (*n* = 16)	7T NHCs (*n* = 21)	3T NHCs (*n* = 61)	*p* value
Male, *n* (%)	19 (100)	4 (25)	11 (52)	56 (92)	<0.001
Mean age at time of scan, years (SD)	40.4 (7.9)	29.9 (19.3)	35.3 (12.7)	30.6 (9.5)	0.009

TBI, head trauma; NHCs, normal healthy controls; SD, standard deviation.

**Table 2.** Fighting History Among Fighters (*n* = 19)

Active vs. retired	Fighting type	No. of professional boxing matches	No. of professional MMA matches
Retired	Boxing	56	0
Retired	Boxing	41	0
Retired	Boxing	47	0
Retired	MMA	0	33
Retired	Boxing and MMA	9	49
Retired	MMA	0	35
Retired	MMA	0	17
Retired	Boxing	38	0
Active	Boxing	29	0
Active	MMA	0	1
Retired	MMA	0	8
Retired	Boxing	17	0
Retired	Boxing	29	0
Retired	MMA	0	52
Retired	Boxing	14	0
Active	Boxing	11	0
Retired	Boxing	27	0
Active	Boxing	5	0
Retired	Boxing	42	0

MMA, mixed martial arts.

### Inter-reader agreement

The two readers agreed on the presence/absence of WMCs for 88% (84 of 95) of participants who underwent 3T MRI (Cohen's kappa, 0.76) and for 93% (51 of 55) of participants who underwent 7T MRI (Cohen's kappa, 0.79). Median absolute discrepancy between the two readers in terms of the total number of WMCs identified was 0 with 3T MRI (interquartile range [IQR]: 0, 2) and 1 with 7T MRI (IQR: 0, 2).

The two readers agreed on the presence/absence of CMHs for 96% (91 of 95) of participants who underwent 3T MRI (Cohen's kappa, 0.76) and for 96% (54 of 56) of participants who underwent 7T MRI (Cohen's kappa, 0.88). Median absolute discrepancy between the two readers in terms of the total number of CMHs identified was 0 with both 3T and 7T MRI (IQR: 0, 0).

### Comparison of 7 Tesla and 3 Tesla magnetic resonance imaging in detecting white matter changes and cerebral microhemorrhages among fighters and patients with head trauma

**FIG. 1.**
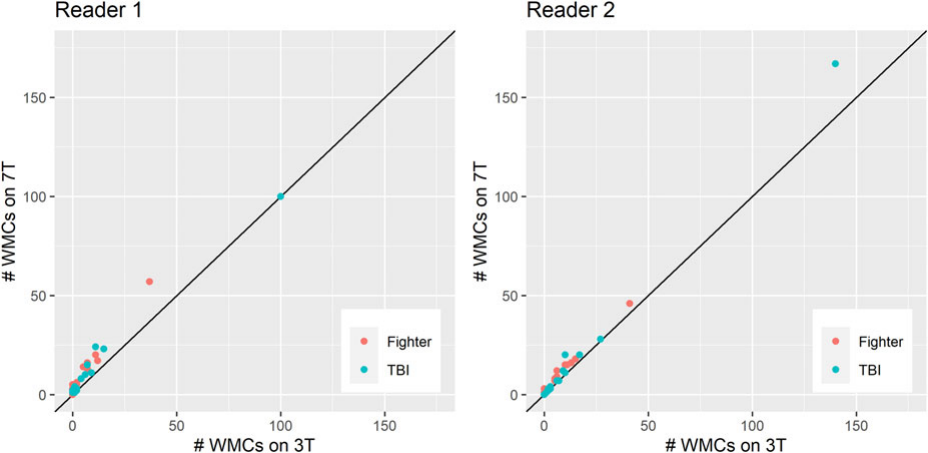
Number of white matter changes (WMCs) detected on 3T MRI versus on 7T MRI among fighters (*n* = 19) and patients with TBI (*n* = 15). MRI, magnetic resonance imaging; T, Tesla; TBI, head trauma.

For both readers and in both patient subgroups (fighters and patients with TBI), the number of WMCs detected was greater with 7T MRI than with 3T MRI (Table 3; Fig. 1). On average, two more WMCs per participant were found with 7T MRI than with 3T MRI (95% confidence interval: 1, 3). For both readers and in both patient subgroups, there was no significant difference in the number of CMHs detected with 7T MRI versus with 3T MRI (Table 3).

**Table 3.** Comparison of 3T and 7T MRI in Detecting WMCs and CMHs in Fighters and Patients With TBI

Nos. of WMCs and CMHs	Fighters (*n* = 19)			Patients with TBI (*n* = 16*^a^*)		
	3T	7T	*p*	3T	7T	*p* value
Reader 1						
Median no. of WMCs [IQR] (range)	1 [0, 6] (0, 37)	3 [1, 14] (0, 57)	<0.001	4 [1, 8] (0, 100)	8 [2, 13] (1, 100)	0.002
Median no. of CMHs [IQR] (range)	0 [0, 0] (0, 2)	0 [0, 0] (0, 2)	0.999	0 [0, 0] (0, 30)	0 [0, 1] (0, 30)	0.181
Reader 2						
Median no. of WMCs [IQR] (range)	3 [1, 8] (0, 41)	3 [2, 14] (0, 46)	<0.001	6 [2, 10] (0, 140)	7 [2, 16]	0.008
Median no. of CMHs [IQR] (range)	0 [0, 0] (0, 8)	0 [0, 0] (0, 9)	0.371	0 [0, 0] (0, 40)	0 [0, 1] (0, 45)	0.181

^a^ Only 15 patients with TBI were assessed for WMCs.

T, Tesla; MRI, magnetic resonance imaging; WMCs, white matter changes; CMHs, cerebral microhemorrhages; TBI, head trauma; IQR, interquartile range.

### Comparison of 3 Tesla magnetic resonance imaging findings among participant groups

For both readers, the number of WMCs detected on 3T MRI tended to be higher among fighters than among NHCs. For reader 2, the number of CMHs was also higher among fighters than among NHCs. There was no statistically significant difference in the number of WMCs or CMHs detected in fighters and patients with TBI (Table 4).

**Table 4.** Detection of WMCs and CMHs with 3T MRI in Fighters, NHCs, and Patients With TBI

Nos. of WMCs and CMHs	Fighters (*n* = 19)	3T NHCs (*n* = 61)	Patients with TBI (*n* = 16*^a^*)	*p* value^*^	*p* value^**^
Reader 1					
Median no. of WMCs [IQR] (range)	1 [0, 6] (0, 37)	0 [0, 0] (0, 12)	4 [1, 8] (0, 100)	0.004	0.166
Median no. of CMHs [IQR] (range)	0 [0, 0] (0, 2)	0 [0, 0] (0, 2)	0 [0, 0] (0, 30)	0.054	1.000
Reader 2					
Median no. of WMCs [IQR] (range)	3 [1, 8] (0, 41)	0 [0, 0] (0, 15)	6 [2, 10] (0, 140)	<0.001	0.229
Median no. of CMHs [IQR] (range)	0 [0, 0] (0, 8)	0 [0, 0] (0, 1)	0 [0, 1] (0, 40)	0.027	0.641

^*^ Fighters versus NHCs.

^**^ Fighters versus patients with TBI.

^a^ Only 15 patients with TBI were assessed for WMCs.

WMCs, white matter changes; CMHs, cerebral microhemorrhages; T, Tesla; MRI, magnetic resonance imaging; NHCs, normal healthy controls; TBI, head trauma; IQR, interquartile range.

### Comparison of 7 Tesla magnetic resonance imaging findings among participant groups

For both readers, the numbers of WMCs overall and subcortical WMCs detected on 7T MRI were higher among fighters than among NHCs. There was no statistically significant difference between fighters and patients with TBI. There was also no statistically significant difference in the number of periventricular WMCs or in the number of CMHs detected among the participant groups on 7T MRI (Table 5).

**Table 5.** Detection of WMCs and CMHs with 7T MRI in Fighters, NHCs, and Patients With TBI

Nos. of WMCs and CMHs	Fighters (*n* = 19)	7T NHCs (*n* = 21)	Patients with TBI (*n* = 16*^a^*)	*p* value^*^	*p* value^**^
Reader 1					
Median no. of WMCs overall [IQR] (range)	3 [1, 14] (0, 57)	1 [0, 3] (0, 12)	8 [2, 13] (1, 100)	0.016	0.374
Median no. of subcortical WMCs [IQR] (range)	3 [1, 8] (0, 19)	1 [0, 2] (0, 12)	4 [1, 12] (0, 100)	0.017	0.613
Median no. of periventricular WMCs [IQR] (range)	0 [0, 1] (0, 38)	0 [0, 0] (0, 4)	0 [0, 2] (0, 4)	0.315	0.755
Median no. of CMHs [IQR] (range)	0 [0, 0] (0, 2)	0 [0, 0] (0, 1)	0 [0, 1] (0, 30)	0.252	0.207
Reader 2					
Median no. of WMCs overall [IQR] (range)	3 [2, 14] (0, 46)	1 [0, 3] (0, 15)	7 [2, 16] (0, 167)	0.004	0.497
Median no. of subcortical WMCs [IQR] (range)	3 [1, 12] (0, 37)	1 [0, 2] (0, 15)	6 [2, 16] (0, 167)	0.007	0.577
Median no. of periventricular WMCs [IQR] (range)	0 [0, 0] (0, 9)	0 [0, 0] (0, 1)	0 [0, 1] (0, 3)	0.266	0.591
Median no. of CMHs [IQR] (range)	0 [0, 0] (0, 9)	0 [0, 0] (0, 1)	0 [0, 1] (0, 45)	0.120	0.289

^*^ Fighters versus NHCs.

^**^ Fighters versus patients with TBI.

^a^ Only 15 patients with TBI were assessed for WMCs.

WMCs, white matter changes; CMHs, cerebral microhemorrhages; T, Tesla; MRI, magnetic resonance imaging; NHCs, normal healthy controls; TBI, head trauma; IQR, interquartile range.

### Distribution of white matter changes within each group on 7 Tesla magnetic resonance imaging

For both readers, the number of WMCs detected with 7T among fighters was higher in the subcortical region than in the periventricular region (reader 1, *p* = 0.043; reader 2, *p* = 0.001). Similarly, among patients with TBI, the number of WMCs detected with 7T was higher in the subcortical region than in the periventricular region (reader 1, *p* = 0.020; reader 2, *p* = 0.003).

### Correlation between white matter changes and patient characteristics among fighters

For both readers, no significant associations were observed between the number of WMCs detected and various patient characteristics (Table 6).

**Table 6.** Spearman's Rank Correlation (*r_S_*) Between WMCs and Various Characteristics in Fighters (*n* = 19)

Characteristic	WMCs with 3T MRI		WMCs with 7T MRI	
	r_S_	*p* value	r_S_	*p* value
Reader 1				
Presence of neurofilament light protein^a^	–0.15	0.589	–0.05	0.845
Verbal memory score	–0.12	0.635	0.10	0.689
Psychomotor speed score	–0.25	0.306	–0.08	0.741
Reaction time	0.15	0.550	0.14	0.578
Patient Health Questionnaire score	0.05	0.827	0.06	0.802
Barratt Impulsiveness Scale score	0.16	0.500	0.15	0.548
No. of professional boxing fights	0.34	0.153	0.27	0.257
No. of professional MMA fights	–0.06	0.821	–0.06	0.808
Reader 2				
Presence of neurofilament light protein^a^	–0.02	0.931	0.03	0.922
Verbal memory score	0.04	0.874	0.03	0.889
Psychomotor speed score	–0.15	0.530	–0.03	0.902
Reaction time	0.06	0.809	0.14	0.561
Patient Health Questionnaire score	0.14	0.580	0.08	0.736
Barratt Impulsiveness Scale score	0.06	0.808	0.03	0.914
No. of professional boxing fights	0.21	0.386	0.25	0.293
No. of professional MMA fights	0.05	0.834	–0.04	0.855

^a^ Only 16 fighters underwent the neurofilament light protein test.

WMCs, white matter changes; MMA, mixed martial arts; T, Tesla; MRI, magnetic resonance imaging.

## Discussion

In this retrospective study, we found that more WMCs were detected on 7T MRI than on 3T MRI regardless of participant status (fighter, patient with TBI, or NHC). We also found that there was a significantly higher number of WMCs in fighters and in patients with TBI than in NHCs on both 3T and 7T MRI.

Previous studies of WMCs in fighters and patients with TBI were conducted using 1.5T or 3T MRI and demonstrated conflicting results. This may reflect limitations in the ability to detect WMCs at these relatively lower field strengths. In this study, we found that 7T MRI appears to be more sensitive to WMCs, perhaps attributable to smaller voxel size and better signal-to-noise ratio. If larger studies can confirm these findings, this field strength should be considered for the assessment of focal white matter track damage. Because 7T MRI was recently approved by the U.S. Food and Drug Administration for clinical applications, expanding its use should be relatively easy.

Previous diffusion tensor imaging (DTI) studies have demonstrated increased diffusivity in different white matter tracts^18,19^ in fighters or patients with TBI versus in NHCs; however, this is a subtly different result than the increased WMCs we observed in the current study. Changes observed on DTI are broad and involve much larger white matter tracts in the brain, whereas the increased WMCs observed in the current study were more focal and could be appreciated on conventional imaging. However, it is uncertain whether the increase in WMCs we observed in this study represents focal injury to white matter tracts, a sign of neuronal damage that can be observed on conventional MRI, or other etiologies. Given the small numbers in our study, we cannot exclude other possibilities for increased WMCs in fighters and patients with TBI, such as migraines or confounding lifestyle activities with risks that could also lead to WMCs. Further evaluations are needed in larger patient populations with more balanced groups in regard to age and sex, as well as longer follow-up and pathological correlation.

In this study, no differences were noted between 7T and 3T MRI in the number of CMHs detected, which suggests that 7T MRI may have limited additional value in the evaluation for CMHs. This may be attributable to the strong intrinsic sensitivity of susceptibility-weighted sequences to blood products, with avid blooming that can be discerned even at 3T and is not greatly amplified at 7T.

We also found that WMCs detected on 7T MRI were more frequently observed in the subcortical region than in the periventricular region for both fighters and patients with TBI. This finding agrees with the results of previous studies, which demonstrated that patients with mild TBI tend to have lesions closer to the gray matter/white matter junction.^7,8,10^

No significant correlations were observed between the number of WMCs and neuropsychological testing scores in fighters. Additionally, the number of WMCs in fighters did not correlate with plasma neurofilament light protein levels. The small sample size could have contributed to these negative results. Moreover, the level of neurofilament light protein has previously been shown to correlate with more acute TBI rather than with chronic exposure to RHI.^20^

This study had several limitations. The sample sizes for the cohorts were small, with only 19 fighters, 16 patients with TBI, twenty-one 7T NHCs, and sixty-one 3T NHCs. Moreover, the groups were not balanced in regard to age and sex, which are potential confounders. Although we had extensive medical and psychosocial history for the fighters in this analysis, we did not have this information for the NHCs or patients with TBI. Additionally, one reader (J.L.) was not blinded to patient group (fighter, patient with TBI, or NHC) when assessing the number of WMCs and CMHs, but this is likely a minor limitation given that the other reader (S.J.) was blinded and there was still good correlation between the readers. The readers were also not blinded to magnet strength (3T vs. 7T); such blinding is not possible because of the unique image characteristics that distinguish the field strengths.

## Conclusion

This study demonstrated that 7T MRI can detect more WMCs than 3T MRI. In addition, fighters and patients with TBI were found to have more WMCs than NHCs on both 3T and 7T MRI. The number of CMHs did not differ significantly between fighters/patients with TBI and NHCs, and no differences were observed between 7T and 3T MRI in the number of CMHs detected. Further studies in larger patient populations are needed, which should be easily achievable as the use of 7T MRI becomes more prevalent clinically.

## Abbreviations Used

CMHs: cerebral microhemorrhages
CTE: chronic traumatic encephalopathy
DTI: diffusion tensor imaging
FLAIR: fluid-attenuated inversion recovery
GRAPPA: generalized autocalibrating partially parallel acquisitions
IRB: institutional review board
MRI: magnetic resonance imaging
NHCs: normal healthy controls
RHIs: repetitive head impacts
SWI: susceptibility-weighted imaging
T: Tesla
TBI: head trauma
TE: echo time
TI: inversion time
TR: repetition time
WMCs: non-specific white matter changes
